# Co-founding ant queens prevent disease by performing prophylactic undertaking behaviour

**DOI:** 10.1186/s12862-017-1062-4

**Published:** 2017-10-13

**Authors:** Christopher D. Pull, Sylvia Cremer

**Affiliations:** 1 IST Austria (Institute of Science and Technology Austria), Am Campus 1, 3400 Klosterneuburg, Austria; 2 Present address: School of Biological Sciences, Royal Holloway University of London, Egham, TW20 0EX UK

**Keywords:** Host-pathogen interactions, Social immunity, Disease defence behaviour, Entomopathogenic fungus, Pleometrosis

## Abstract

**Background:**

Social insects form densely crowded societies in environments with high pathogen loads, but have evolved collective defences that mitigate the impact of disease. However, colony-founding queens lack this protection and suffer high rates of mortality. The impact of pathogens may be exacerbated in species where queens found colonies together, as healthy individuals may contract pathogens from infectious co-founders. Therefore, we tested whether ant queens avoid founding colonies with pathogen-exposed conspecifics and how they might limit disease transmission from infectious individuals.

**Results:**

Using *Lasius niger* queens and a naturally infecting fungal pathogen *Metarhizium brunneum,* we observed that queens were equally likely to found colonies with another pathogen-exposed or sham-treated queen. However, when one queen died, the surviving individual performed biting, burial and removal of the corpse. These undertaking behaviours were performed prophylactically, i.e. targeted equally towards non-infected and infected corpses, as well as carried out before infected corpses became infectious. Biting and burial reduced the risk of the queens contracting and dying from disease from an infectious corpse of a dead co-foundress.

**Conclusions:**

We show that co-founding ant queens express undertaking behaviours that, in mature colonies, are performed exclusively by workers. Such infection avoidance behaviours act before the queens can contract the disease and will therefore improve the overall chance of colony founding success in ant queens.

**Electronic supplementary material:**

The online version of this article (10.1186/s12862-017-1062-4) contains supplementary material, which is available to authorized users.

## Background

Behaviour that decreases the probability of an individual acquiring pathogens should confer fitness advantages and be selected for over time [1]. This is because mounting an immune response post-infection can have a severe impact on an animal’s future reproduction and survival, whilst behavioural mechanisms can negate these costs by preventing infection altogether [2–5]. Most often, these behaviours are the avoidance of contagious conspecifics or areas they have contaminated [6]. For example, animals avoid sheltering, interacting and mating with infectious counterparts [7–9], and in humans, disgust at disease-associated stimuli is thought to be adaptive as it should reduce pathogen exposure [10, 11]. Behavioural responses that minimise infection risk are therefore considered an important but less well studied component of a host’s disease defence repertoire [12, 13].

In social insects (ants, termites and some bees and wasps) workers perform collective behaviours, such as grooming, which reduce the per capita risk of infection and result in colony-level disease protection, known as social immunity [14]. However, daughter queens often lack this protection as they typically leave the parental nest and found new colonies without the assistance of workers [14]. The rate of mortality for founding queens is therefore high and many die as a result of disease [15–19]. In some ant species queens found new colonies with other, usually unrelated queens, known as co-founding or pleometrosis [20]. Although co-founding can improve queen survival [15, 21], we suggest it may also increase the queens’ risk of disease if co-foundresses fall sick and become infectious. For example, fungus-infected ants can release millions of new infectious conidiospores after death, which significantly reduce the survival of other colony members [22, 23]. Furthermore, even non-diseased corpses have negative impacts on worker and brood survival if they are not removed from the nest [24]. We therefore suggest that dead and/or infectious co-foundresses could affect the colony founding success of surviving queens. Subsequently, we might expect selection acting on queens to produce behaviours that reduce this risk.

Ant queens can assess the quality (e.g. the size and condition) of their conspecifics and this affects who they co-found with in the laboratory [25]. As social insect queens can also detect pathogens [26–28], they may therefore avoid co-founding with pathogen-contaminated queens to reduce their own infection risk. However, the decision to co-found is influenced by several factors, including nest site availability and the danger of desiccation, which could supersede co-founder choosiness [28–30]. In addition, ant queens may perform behaviours that prevent pathogen transmission from infected co-foundresses, similar to colony founding termites, which have been reported to groom and bury freeze-killed co-founders, thereby reducing subsequent saprophytic microbial growth on their corpses [31]. However, it remains unclear if these corpse-induced responses, known generally as “undertaking behaviours” [32], actually affect disease transmission, as they are expressed immediately following death, whilst the infectious potential of the corpse may only become evident later.

Here, we therefore investigated if and how queens of the black garden ant, *Lasius niger,* are able to reduce their risk of contracting disease from co-foundresses. In *L. niger*, virgin queens leave the parental nest to engage in mating flights. Afterwards, they search for and establish a nest, with co-founding occurring in about 18% of cases (usually in pairs) [33]. Like most ant queens, *L. niger* is a claustral founder, meaning that queens do not leave their nests in the colony foundation stage and, instead of foraging, survive on the metabolism of a finite amount of bodily food reserves. Hence, they can be considered “closed-systems” [34]. During colony founding, *L. niger* queens are naturally infected by several fungal pathogens, including the generalist insect pathogen *Metarhizium brunneum* (CD Pull, unpublished data). These pathogens can be found in abundances of up to 5000 infectious conidiospores/g of soil [35] and insects acquire infections when conidiospores attach to, and penetrate, their cuticle. During the ensuing non-infectious incubation period, the fungus proliferates and eventually causes host death, before sporulating and producing a new generation of infectious conidiospores on the corpse [36].

Using this host-pathogen system, we set up a choice experiment to first investigate how pathogen exposure affects the co-founding decision of queens. We tested if queens avoid co-founding less with pathogen-exposed individuals, compared to sham-treated control queens. Secondly, we studied the behaviour of queens following the death of a co-foundress. We compared how and when queens reacted to both infected and non-infected corpses, and predicted that the queens’ response should differ based on the risk of infection. We then examined whether the behaviours performed by the queens prevent the pathogen from becoming transmissible and infecting the surviving queen.

## Results

### Pathogen exposure and colony co-founding choice

**Fig. 1:**
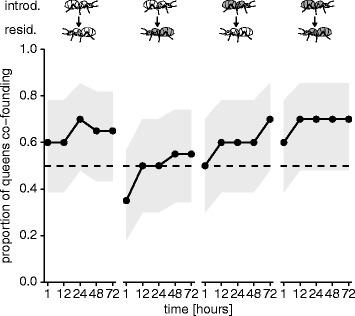
Queen co-founding is independent of pathogen exposure. Introduced queens that were either pathogen-exposed (grey queens) or had received a sham treatment (white queens) were given the choice of founding a colony alone or with a residing queen already present in the nest, which had either been pathogen-exposed (grey queens) or sham-treated (white queens). There was no significant effect of the queen combinations and time on the co-founding decision of queens, revealing that pathogen exposure of neither the residing nor the introduced queen affects colony co-founding choice (points ± grey shading represent proportions ±95% confidence intervals for each time point; 0.5 dashed line added for visual interpretation; GLMM model non-significant). Supporting data in Additional file 1

In our first experiment, we set out to determine how pathogen contamination affects the co-founding decision of queens, from both the perspective of a queen already in the nest and those that may join her. We introduced pathogen-exposed or sham-treated queens to an experimental setup where they could choose to start a nest alone, or with a pathogen-exposed or sham-treated queen already residing in the nest, using a full factorial design. We observed no effect of pathogen-exposure on the likelihood that queens co-found colonies within a 72-h observation period (Fig. 1; overall generalised linear mixed model [GLMM] comprising queen treatment, time and their interaction, *n* = 20 per treatment group, likelihood ratio test (LR) χ^2^ = 4.95, df = 7, *P* = 0.7). On average, 65% of queens across the queen combinations decided to co-found, showing that pathogen exposure does not affect the colony co-founding choice of either the residing or the introduced queen. When co-founding occurred, the introduced queens were present in the residing queens’ chamber in 89% of cases.

### Queen undertaking behaviour

**Fig. 2:**
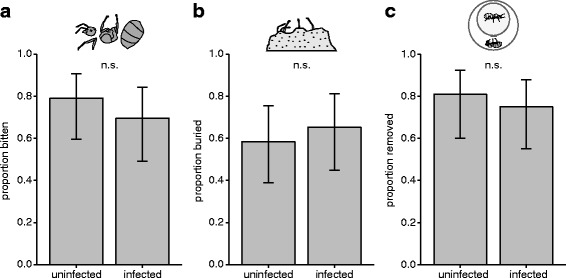
Queens perform prophylactic undertaking behaviours towards co-foundress corpses. Queens encountering non-infected and infected corpses performed several behaviours, namely corpse **a** biting and **b** burying or **c** removal. These behaviours were performed equally towards both infected and non-infected corpses (error bars show ±95% CI; n.s. = non-significant GLM result at α = 0.05). Supporting data in Additional file 2

In a second experiment, we investigated the response of untreated queens to corpses by pairing them with either a pathogen-exposed or sham-treated queen. When pathogen-exposed queens began dying of infections (median survival time = 6 days), we started freeze-killing the sham-exposed queens to test if the untreated queens react differently to infected and non-infected corpses. Queens were kept in either closed nests (a single chamber) or open nests (single chamber with an exit hole that opens into an arena) that contained dried plaster particles as nesting material. We observed the queens performing three undertaking behaviours towards the corpses of both infected and non-infected corpses. In closed nests, 74% of the queens dismantled the corpses by biting them to remove the limbs and break up the body segments and, in 62% of the cases, queens buried the corpses with the plaster particles from the nest. These behaviours were performed equally towards infected and non-infected corpses, with no significant differences in the occurrence of biting (Fig. 2a; infected: 16/23, non-infected: 19/24; GLM, LR χ^2^ = 0.57, df = 1, *P* = 0.45) or burial observed (Fig. 2b; infected: 15/23, non-infected: 14/24; GLM, LR χ^2^ = 0.24, df = 1, *P* = 0.63). These behaviours typically occurred shortly after the death of the co-foundress (median day: biting = 1, burial = 2) and there was no difference in onset regardless of corpse type (biting: infected *n* = 16, non-infected *n* = 17; Mann-Whitney *U* test, *U* = 112*, P* = 0.38; burial: infected *n* = 11, non-infected *n* = 13; Mann-Whitney *U* test, *U* = 64*, P* = 0.68). In open nests, the predominant behaviour, occurring in 78% of the cases, was the removal of the corpse from the nest chamber into the external arena, an undertaking behaviour termed necrophoresis [32]. Again, necrophoresis was performed equally towards both infected and non-infected corpses (Fig. 2c; infected: 18/24, non-infected: 17/21; GLM, LR χ^2^ = 0.23, df = 1, *P* = 0.63), which were also removed from the nest at similar times after co-foundress death (median day: 1; infected *n* = 17, non-infected *n* = 17; Mann-Whitney *U* test: *U* = 152*, P* = 0.81). However, in cases where corpses were left inside the nest (19% of the control and 25% of the pathogen group), queens also performed biting and burial, as in the closed nests (infected: biting occurrence in 1/6 of the replicates, burial in 2/6; non-infected: biting in 2/4, burial in 3/4).

### Fungal outgrowth and onset of undertaking behaviours

**Fig. 3:**
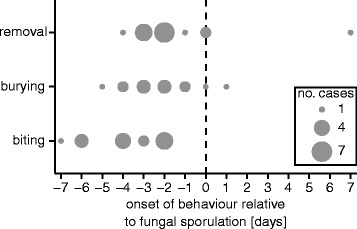
Undertaking behaviours precede corpse sporulation. Queens performed biting, burial and removal before infected corpses sporulated and became infectious. The day behaviours were performed are displayed as points and are relative to fungal sporulation (time point 0; dashed black line), meaning that negative points = before sporulation, and positive = after. The size of the points indicates the number of queens first performing the behaviour on each respective day (‘no. cases’ in legend), meaning that each queens is only represented once. Supporting data in Additional file 3

In 96% of cases (45/47), the corpses of pathogen-exposed queens sporulated and produced new infectious conidiospores, meaning that the behaviours performed by the queens seem generally unable to prevent fungal reproduction. However, queens typically performed undertaking behaviours before the fungus became infectious (% of nests where behaviours occurred before sporulation: biting = 100%; burial = 85%, removal = 82%). Biting, burial and removal all occurred significantly earlier than sporulation (Fig. 3; biting: *n* = 15, Wilcoxon signed-rank test, *Z* = −3.43, *P* = 0.002; burial: *n* = 13, Wilcoxon signed-rank test, *Z* = −2.92, *P* = 0.005; removal: *n* = 17, Wilcoxon signed-rank test, *Z* = −2.6, *P* = 0.01).

### Effect of undertaking behaviours on disease transmission

**Fig. 4:**
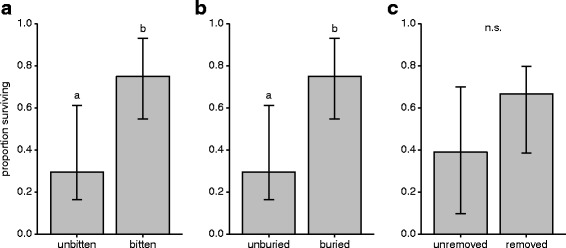
Biting and burial of co-foundress corpses improve queen survival. The number of queens surviving following the death of a co-foundress due to infection, was significantly increased when they performed **a** biting and **b** burial, whereas there was no significant effect for **c** removal (error bars show ±95% CIs given; letters denote significant GLM results at α = 0.05; n.s. = non-significant). Supporting data in Additional file 4

There was no mortality in untreated queens presented with non-infected corpses within the 30 days of the experiment. In contrast, 45% of queens (21/47) in contact with infected corpses died and 95% (20/21) of these sporulated, indicating that the cause of queen death was a *Metarhizium* infection contracted from the corpse. Untreated queen survival was unaffected by the duration that they spent with pathogen-exposed queens before these queens started dying (GLM, LR χ^2^ = 0.65, df = 1, *P* = 0.42). However, we found that the undertaking behaviours the queens performed significantly affected their odds of mortality. In cases where infected corpses were inside the nest (closed nests and open nests where removal was not performed), both biting (Fig. 4a; bitten: 12/17, unbitten: 3/12; GLM, LR χ^2^ = 6.1, df = 1, *P* = 0.02) and burial (Fig. 4b; buried: 12/17, unburied: 3/12; GLM, LR χ^2^ = 6.1, df = 1, *P* = 0.02) caused a sevenfold reduction in the odds of the queens dying. Additionally, the removal of infected corpses reduced the odds of a queen dying by threefold, yet this difference was non-significant (Fig. 4c; removed: 11/18, unremoved: 2/6; GLM, LR χ^2^ = 1.41, df = 1, *P* = 0.24). We also tested whether the onset of performing undertaking behaviours affects untreated queen survival, finding that this was not the case (biting: *n* = 16, GLM, LR χ^2^ = 0.21, df = 1, *P* = 0.65; burial: *n* = 12, GLM, LR χ^2^ = 0.4, df = 1, *P* = 0.27; removal: *n* = 16, GLM, LR χ^2^ = 2.17, df = 1, *P* = 0.4).

## Discussion

Our results reveal that ant queens do not avoid founding nests with pathogen-exposed conspecifics (Fig. 1). But, if a co-foundress dies, queens will bite and bury the corpse or remove it from the nest (Fig. 2). These undertaking behaviours occurred shortly after death and, in the pathogen group, before corpses became infectious (Fig. 3). Importantly, we found evidence to suggest that the biting and burying of corpses improved queen survival (Fig. 4).

Although ant queens risk contracting infections from pathogen-exposed co-foundresses, we show that ant queens do not avoid founding colonies with contaminated individuals. This is most likely because social insect queens have to rapidly find and dig a nest before they succumb to desiccation or predation, and it has been postulated that these risks should therefore outweigh spending time assessing co-founders [28, 29]. Moreover, the patchiness of suitable nest sites and the resultant overcrowding likely forces queens to share, regardless of co-foundress quality [25, 29]. As co-founding can improve queen survival, this could explain their general tendency towards founding colonies together rather than alone [15, 20, 21]. However, co-founding was higher (65%) in our experiments than was previously reported in the field for this species (18% [33]). The reason behind this discrepancy remains unknown. In one case, *L. pallitarsus* queens were found to start colonies more readily together in the laboratory when they were with their sisters [37]; however, in the field, founding queens are usually unrelated [20]. In our study, we did not assess the relatedness of queens, but given the size of mating flights in *L. niger* [38] it is expected that founding queens are most often unrelated [16]. Larger queens also prefer to co-found with smaller queens than to found alone, since they will benefit from cooperating whilst maintaining a competitive advantage [25]. We did not measure the size or weight of our queens, but differences in queen size could have resulted in the levels of co-founding we observed.

When a co-foundress died, we observed the surviving ant queens performing the full repertoire of undertaking behaviours that have so far only been described to be performed by workers [32]. In general, when nests were open, queens performed necrophoresis, whilst when they were closed, biting and burial was performed instead. Hence, our results reveal that although ant queens in mature colonies focus on reproduction, they can perform sanitary behaviours during the early, workerless stages of their lifecycle. Interestingly, we observed queens performing undertaking behaviours prophylactically towards both non-infected and infected corpses, which is in line with studies on workers showing that undertaking and self-removal occur in the absence of infection and before disease symptoms appear [32]. Importantly, the onset of these behaviours also preceded the fungus sporulating and becoming infectious. Acting prophylactically may be important as both infected and non-infected corpses can impact the survival of nestmates, e.g. due to the post-mortem growth of infectious and saprophytic microorganisms [22–24, 39]. Moreover, acting early may maximise the likelihood that disease transmission is prevented, as pathogenic fungi usually take 1–2 days to sporulate [39, 40]. The diverse repertoire of undertaking behaviours we observed in queens is probably important given that, at certain times of the year, necrophoresis could be difficult to perform because the ground is too hard for the queens to dig an additional chamber to place the corpse, or to dig her way to the surface of the soil. Additionally, excessive digging can increase queen mortality, presumably because they exhaust the body reserves that they store for sustenance and brood-rearing [41], and it may expose them to further pathogens in the soil. Hence, in the wild, the type of undertaking behaviour performed may be influenced by several factors.

Forty eight per cent of queens that founded colonies with pathogen-exposed individuals contracted infections and died. This mortality could have resulted from cross-contamination that occurred after pathogen-exposed queens were introduced to the nest, i.e. before the fungal conidiospores had adhered to their cuticles and could have been transferred to the untreated queen [42, 43]. Such cross-contamination could occur via contaminated nest surfaces, but is most likely to arise during allogrooming, which *L. niger* queens do not perform [26]. Moreover, such infections are normally low-level and thus non-lethal [42]. In addition, all untreated queens in cases where the pathogen-exposed queens survived (11/58 nests) did not contract lethal infections, suggesting that untreated queens in the other nests most likely contracted infections from the sporulating, infectious corpses. Indeed, they all died only after the sporulation of the co-foundress corpse and previous work has shown that infectious corpses are especially virulent, due the large quantity of conidiospores they release [22, 23, 40].

Given that the queens perform undertaking behaviours on their own, it may not be surprising that they cannot prevent sporulation, which in mature colonies requires the concerted action of multiple workers [40]. Despite this limitation, however, queens that performed biting and burial had improved survival. Whilst further experiments are needed to conclusively prove that this relationship is causal – since fitter queens that can survive infection may be more likely or able to perform these behaviours – comparing the survival of queens performing undertaking to those that do not provides strong correlational evidence that undertaking behaviours reduce the risk of queens contracting lethal infections. We noted from visual inspections that corpses bitten and buried by the queens supported less conidiospore growth than those that were only removed from the nest or single queens kept in isolation. It has been suggested that biting can desiccate corpses and create unfavourable conditions for the pathogen [14]. In our experiment, humidity was optimal for fungal growth throughout, so that biting the corpses into smaller pieces might give the additional benefit of limiting the amount of nutrients available for the fungus and result in a reduction in the total amount of new infectious conidiospores produced from corpses. As the probability of mortality from a *Metarhizium* infection is directly linked to exposure dose [44, 45], biting may therefore improve the survival of the co-founding queen by reducing how many infectious propagules she is confronted with from the sporulating corpse.

Corpses that have been dismantled through biting may also be buried more efficiently and both behaviours were usually performed together. Burial is an effective undertaking behaviour as it physically separates the infectious corpse from susceptible insects [31, 46, 47]. Thus, queens that perform this behaviour should have a lower risk of contracting an infection than queens who did not. Whether buried corpses will be excavated as the colony grows is unknown. However, areas of the nest containing buried corpses are avoided by termites [46] and the infectivity of *Metarhizium* conidiospores decreases with time [48, 49], making it less likely that co-foundress corpses cause infections after the first workers emerge.

In contrast to biting and burial, necrophoresis did not significantly enhance queen survival. This is surprising because, as in mature ant [24, 39, 50, 51] and honeybee [52] colonies, this behaviour isolates the corpse and should prevent disease transmission. The absence of a statistically significant difference, despite a threefold improvement of survival in the queens performing the behaviour, could have been driven by the low number of queens not performing the behaviour (6/24), which likely reduced our statistical power. However, it is also possible that the queens still interacted with the corpses after removal. Indeed, both ant [27] and termite queens [28] appear to be attracted to conidiospores in their environment and removed corpses were typically left unbitten and unburied, so contact with the corpses would lead to disease transmission and thus infection.

## Conclusion

In this study, we have characterised the undertaking behaviours of colony founding ant queens. Whilst co-founding queens neither avoid pathogen-exposed individuals (this study) nor perform any sanitary care towards them [26], here we have shown that they are indeed able to perform undertaking behaviours, which are typically considered worker-tasks in mature colonies. In the absence of workers, the queens therefore act to prophylactically protect themselves from disease. Although several studies have demonstrated the immunological capabilities of founding ant queens [26, 53–56], avoiding infection may be particularly important because they are ‘closed systems’, surviving solely on the breakdown of fat and muscle reserves until the first workers emerge and begin foraging [34, 57]. Queens must therefore balance their limited resources and investment into immunity can cause trade offs with other life history traits [26, 56]. For example, queens exposed to *Metarhizium* can survive infection by resisting the pathogen, however, they subsequently produce fewer workers than healthy individuals [26]. Smaller colonies are at a competitive disadvantage and are more likely to fail before new sexual offspring are produced [20]. Although protected by workers in mature colonies, social insect queens, like other animal taxa [6], should therefore be under selection to evolve both immunological [2, 53–56] and behavioural (this study, [31]) adaptations against disease, in order to survive the risky colony founding stage. Hence, performing undertaking behaviours to improve survival should therefore increase a queen’s chances of successfully founding a colony that then develops into a mature nest.

## Methods

### Queen collection and maintenance

We collected hundreds of mated queens in July 2013 and 2014 from the IST Austria campus, Klosterneuburg, Austria. Queens were returned to the laboratory in plastic boxes containing damp tissue paper until they were used in experiments, which was within one day of collection. Mortality during this time period was minimal and appeared to result from damage caused during the mating flight or collection, e.g. because of missing limbs or damaged abdomens. Queens collected during mating flights can have natural infections (ca. 1% of queens have fungal infections; C.D. Pull, unpublished data), however, in none of our experiments did we observe any pathogenic growth other than *Metarhizium*. No food was provided to queens in any of the experiments as they survive solely on the breakdown of muscle and fat reserves during colony founding [34]. Queens were chosen for each experiment/treatment group haphazardly.

### Fungal pathogen

We used the species *M. brunneum* (strain KVL-03-143), collected from and grown on sabaroud dextrose agar plates before each experiment for approximately two weeks, until plates were fully sporulating. Conidiospores were suspended in autoclaved 0.05% Triton-X (in water) and washed three times by centrifuging the conidiospores (3000 g for 5 min), pouring away the supernatant, and re-suspending them again 0.05% Triton-X. We confirmed the viability of the conidiospores by plating out 100 μl of the conidiospore suspension onto sabouraud dextrose agar plates and checking the number of conidiospores germinating after 18 h (always >90%).

### Queen pathogen exposure

Queens were exposed to the fungal suspension or autoclaved Triton-X as a sham-exposure, by gently restraining them with soft forceps and pipetting 0.5 μl of the fungal suspension or Triton-X onto their thorax. Queens were then placed onto filter paper to remove excess liquid and allowed to dry alone for several minutes before being added to experiments. For all experiments, we applied a droplet of 0.5 μl (2 × 10^7^ conidiospores ml^−1^), which causes high mortality in queens (30/35 queens) kept alone for 30 days (median survival time = 6 days).

### Experiment 1: Effect of pathogen exposure on colony co-founding choice

All queens were individually colour-marked (paint “Uni Posca”) on one of their abdominal segments to differentiate the pathogen-exposed and sham-treated queens. We set up plastic boxes (10 × 3.5 cm; Bock GmbH & Co. KG) comprising three equally sized chambers and a transparent lid. The middle chamber was uncovered and had no substrate, whereas the two chambers on either side were covered in red transparent foil (to reduce light entering the chamber whilst allowing observations of the ants) and had a damp plaster substrate. A hole (5 mm Ø) in the walls of the middle chamber connected it to the two adjacent chambers, allowing the ant queens to move freely between them.

Into one of the plastered and darkened chambers, we placed either a sham-treated or pathogen-exposed queen, which we termed the “residing queen”. After allowing her time to settle (1 h), we then introduced a second queen to the middle chamber, which was either sham-treated or pathogen-exposed, and termed the “introduced queen”. We varied which of the two darkened chambers the first queen was placed into in case there was a directional bias towards one of the chambers. In the field, queens choose to co-found or not within the first few hours following a mating flight and about half of queens are underground within 40 min of landing [29, 58], so our set up of allowing the residing queens 1 h to settle approximates field conditions.

Thus, we had four experimental groups (i) a sham-treated queen introduced to a nest with a sham-treated residing queen (ii) a sham-treated queen introduced to a nest with a pathogen-exposed residing queen (iii) a pathogen-exposed queen introduced to a nest with a residing sham-treated queen and (iv) a pathogen-exposed queen introduced to a nest with a residing pathogen-exposed queen (*n* = 20 in all cases). Following the introduction of the second queen, we observed the locations of queens after 1, 12, 24, 48 and 72 h. We stopped observations at 72 h as ~75% of queens had produced eggs and queens started dying from the fungal exposure after this point. The experiment was run at 23 °C and 70% humidity, under continuous light, to encourage queens to choose one of the darkened chambers, as opposed to remaining in the middle chamber. All queens in this experiment were collected in 2013.

### Experiment 2: Queen behaviour towards co-foundress corpses and disease transmission

We placed single, unpainted and untreated queens into petri dishes (Ø = 3.5 cm) filled with damp plaster that contained a rectangular cavity measuring 1 cm × 3.5 cm, to mimic the small chambers queen’s construct when founding a colony. Each chamber contained 1 g of loose plaster particles as a nest material. The lids of the dishes were covered with red transparent foil to keep the chamber darkened. We termed these dishes “closed nests”. Half of the dishes remained closed nests, whilst the other half were placed into a second, larger dish (Ø = 9 cm) with a plaster substrate. A small hole (Ø = 5 mm) in the side of the small dish allowed the queen access to this external arena, to create an “open nest”. We then added a second, paint-marked queen (allowing us to distinguish her from the untreated queen) to each dish in both the closed and opens nests, which was either sham-treated or pathogen-exposed.

We monitored the survival of pathogen-exposed queens on a daily basis and noted when they died. So that we could compare the behaviours of naïve queens towards infected and non-infected queens when they died, we removed and froze sham-treated queens on the days that pathogen-exposed queens died to create non-infected dead queens (mean day of death ± standard deviation: pathogen-killed = 6.4 ± 1.7; freeze-killed = 6.5 ± 1.9). These queens were frozen for 5 min at −80 °C, before being added back to the dishes with the surviving queens. We did not freeze pathogen-exposed queens when they died in case this affected the outgrowth of the fungus. However, freezing as a method of killing has been used to study undertaking behaviour in other ant species, termites and bees, and elicits typical undertaking responses in nestmates [31, 50, 59, 60]; however, infected corpses may be more attractive or elicit more rapid undertaking behaviour [39, 61]. The pathogen-exposed queens died in the majority of nests (47 out of a total of 58 that we set up) and those where they did not were not included in the analysis. There was no difference between the survival of untreated queens in the control group when they were kept in closed or open nests (100% survival in both cases), meaning that nest type did not affect their mortality. Overall, once the queens were killed by the pathogen or freezing, we had four treatment groups: (i) untreated queens in closed nests with the corpse of a pathogen-exposed queen (*n* = 23); (ii) untreated queens in closed nests with the corpse of a sham-treated queen (*n* = 24); (iii) untreated queens in open nests and the corpse of a pathogen-exposed queen (*n* = 24); (iv) untreated queens in open nests and the corpse of a sham-treated queen (*n* = 21).

On a daily basis, each nest was inspected visually for several minutes to record the presence or absence of a behavioural response of untreated queens to corpses, as well as when sporulation occurred. Burial was recorded when queens had covered the corpses in plaster or had built a ‘wall’ that separated the untreated queen from the corpse, whilst biting was defined as the removal of limbs and/or body segments. In addition, we recorded the survival of the untreated queens and, when they died, if sporulation occurred on their corpse, which we always identified to be *Metarhizium*. In a few cases, the exact timing of the occurrence of the behaviour (2/36 for biting, 5/31 for burial, 1/35 for removal), or sporulation (1/45) was missed. Exact sample sizes per test are provided in the results section. The experiment was run at 23 °C and 70% humidity, under a 12 h light:dark schedule, though because dishes were covered in red foil, the closed nests and the smaller chamber in the open nests were always darkened, mimicking the dark chambers in which queens reside. The duration of the experiment, from pathogen exposure to the final inspection for fungal growth, was 30 days. All queens in this experiment were collected in 2014.

### Data analysis

All statistical data analysis was carried out using R version 3.3.2 [62]. We analysed the colony co-founding choice of queens using a generalised linear mixed model (GLMM; ‘lme4’ R package [63]), including chamber choice as a logistic response and a predictor for when the introduced queen was pathogen-exposed, a predictor for when the residing queen was pathogen-exposed, a predictor for time (*z*-transformed) and a three-way interaction between all three predictors to assess if co-founding choice differs over time. To control for the repeated observation of the same replicate, a random intercept was included for each replicate, and their individual differences over time were explicitly modelled by including random slopes for each individual. General linear models (GLMs) with binomial error terms and logit-link functions were used to compare the behaviour of queens towards infected and non-infected corpses, including the presence/absence of the behaviour (biting, burial or removal) as the response and the type of corpse (infected or non-infected) as a predictor and day (log transformed to achieve normality) of treated queen death as a covariate (since queens died on different days). However, as the covariate was always non-significant (biting: LR χ^2^ = 0.28, df = 1, *P* = 0.6; burial: LR χ^2^ = 0.28, df = 1, *P* = 0.6; removal: LR χ^2^ = 0.98, df = 1, *P* = 0.32), we removed it from the models to gain better estimates for the remaining predictor. In these models, we analysed open and closed nests separately, given that there were clear differences in the types of behaviours performed between nest types. Mann-Whitney *U* tests were used to test for differences between the day of onset of undertaking behaviours between infected and non-infected corpses. Wilcoxon signed-rank tests were used to compare the days that the undertaking behaviours and fungal sporulation occurred, and to control for multiple testing, we corrected the resulting *P* values using the Benjamini-Hochberg procedure to protect against a false discovery rate of 0.05% [64]. Adjusted *P* values are reported. The survival of queens performing different behaviours was analysed using GLMs with binomial error terms and logit-link functions that included mortality of the untreated queen as the response and the presence/absence of the behaviour (biting, burial or removal) as the predictor. Again, we controlled for multiple testing by correcting the *P* values from these models using the Benjamini-Hochberg procedure [64]. We analysed whether the onset of the behaviour affected untreated queen survival by including the mortality of the untreated queen as the response and the day the behaviour was performed, relative to queen death, as the predictor. For these models, we log(x + 1) transformed day to achieve normality, and corrected the resulting *P* values using the Benjamini-Hochberg procedure. We also tested whether, overall, the duration that untreated queens (including both those from open and closed nests) were with the pathogen-exposed queens before they died affected survival in the same way, again, log(x + 1) transforming day. We ensured all data fit the assumptions of the models (i.e. normality of predictors, multicollinearity, Cook’s distance, dffits, dfbetas and leverage) and overall model significance, plus the effect of predictors, were tested by comparing full models to nested null and reduced models, respectively, where all predictors present occur in the full model (except those being tested), using likelihood ratio tests. All graphs were made using the ‘ggplot2’ R package [65].

## Additional files

Additional file 1: Queen association dataset. Data arising from experiment 1, testing how pathogen exposure affects the co-founding decisions of *Lasius niger* ant queens. (XLS 60 kb)

Additional file 2: Behavioural comparison dataset. Data arising from experiment 2, comparing the undertaking behaviours of *Lasius niger* queens performed toward infected and non-infected corpses. (XLS 41 kb)

Additional file 3: Fungal outgrowth and behavioural onset dataset. Data arising from experiment 2, showing the day of fungal outgrowth of *Metarhizium* from corpses and the onset of undertaking behaviours performed by *Lasius niger* queens. (XLS 23 kb)

Additional file 4: Effect of undertaking dataset. Data arising from experiment 2, testing the effect of undertaking behaviours performed by *Lasius niger* queens on disease transmission from corpses and queen survival. (XLS 50 kb)
